# The influence of the team in conducting a systematic review

**DOI:** 10.1186/s13643-017-0548-x

**Published:** 2017-08-01

**Authors:** Lesley Uttley, Paul Montgomery

**Affiliations:** 1 School of Health and Related Research, University of Sheffield, Regent Court, 30 Regent Street, Sheffield, S1 4DA UK; 2 Department of Social Policy and Social Work, University of Birmingham, Muirhead Tower, Edgbaston, Birmingham, B15 2TT UK

**Keywords:** Research, Bias, Team, Meta-bias, Affiliation, Reviewer

## Abstract

There is an increasing body of research documenting flaws in many published systematic reviews’ methodological and reporting conduct. When good systematic review practice is questioned, attention is rarely turned to the composition of the team that conducted the systematic review. This commentary highlights a number of relevant articles indicating how the composition of the review team could jeopardise the integrity of the systematic review study and its conclusions. Key biases require closer attention such as sponsorship bias and researcher allegiance, but there may also be less obvious affiliations in teams conducting secondary evidence-syntheses. The importance of transparency and disclosure are now firmly on the agenda for clinical trials and primary research, but the meta-biases that systematic reviews may be at risk from now require further scrutiny.

## Main text

Systematic reviews benefit from team working, and co-production is an essential part of high-quality research synthesis and healthcare decision [1, 2]. However, despite their reputation as transparent and rigorous products, they are influenced by the people who conduct them and this in turn could affect the resulting conclusions. A review team may comprise experienced systematic reviewers, information specialists, statisticians, and content experts, or, as no licence is required to conduct a systematic review, the review team may include none of these specialties. In this case, it may be wise to consider who is conducting the systematic review and why. The number of systematic reviews indexed in MEDLINE has increased threefold over the last decade [3] indicating a steady spread in their employment globally. However, as in primary research, [4] systematic reviews can vary hugely in their reporting quality or their methodological quality [5–7]. Indeed, many systematic reviews are receiving increasing criticism for failing to live up to their reputation as high-quality, well-conducted pieces of research [8, 9]. They can be susceptible to bias, for example, when reviews are conducted by people who have a stake in the conclusions (researcher allegiance) [10, 11]. Alternatively, reviews could be conducted carelessly, in the chosen methods of meta-analysis or study selection (meta-bias) [12], or by failing to report research misconduct even when identified [13]. If flawed systematic reviews continue to be published, they risk losing their eminent position in the evidence hierarchy; therefore, closer examination of who might be conducting them, and how the output can be affected, is warranted.

Ordinarily, systematic reviews are conducted to summarise evidence objectively for an intervention’s effectiveness to decision-makers or to highlight where evidence to support an intervention is lacking. Funding bodies often require applicants of new primary research to reference an existing systematic review or to review the relevant evidence prior to commencing new clinical trials. A less worthy motive for conducting a systematic review could be simply to get something published, [14] whether affiliated to the intervention or not. But a more troubling motive is that systematic reviews may be conducted and published by people who have a clear stake in publishing positive results. How can we know if the authors of a systematic review are affiliated to the review question? Despite the existence of guidelines and reporting standards for systematic reviews, it is not currently a requirement to declare the motives of the reviewers, i.e., who is behind the origination of the review question (except for sponsored work) and whether the same people who set the review question are also involved with answering the review question. Information about whether the research question originated from outside the review team, such as through a commissioned call, are not currently deemed as relevant to potential assessment of bias in systematic reviews. Recent research by Borah et al. [15] indicates that funded reviews took significantly longer to complete and involved more authors and team members than studies which did not report funding. A longer timeframe could be indicative of increased rigour in funded reviews. The same study authors also noted from inspection of 195 published reviews identified from the PROSPERO database that the number of “team members” is also discrepant from the number of authors of resulting publications. Their findings indicate that by the time the review reaches publication, there are more authors than people initially registered. How might systematic reviews accrue authors from protocol to final publication and should this be of concern?

Whilst explicit and transparent a priori methods are a hallmark of systematic reviews, in practice, they can be an iterative process, with movement back and forth between phases depending on the results obtained at each step. Despite this flexibility, systematic review authors are responsible for ensuring transparency. A systematic review protocol aims to ensure that necessary deviations from planned methods are evidenced and ideally discussed by the review team. However, even when methods are stated a priori in systematic review protocols, deviations from the protocol, whether justified or not, may be seldom reported in the final publication, as highlighted by Silagy et al. [16] who found 43 out of a sample of 47 published Cochrane reviews had undergone a major change compared with the most recently published protocol. Moreover, Kirkham et al. [17] found discrepancies between protocol and published reviews in a sample of 288 Cochrane reviews in the reported primary outcome. The authors highlight this potential bias in the systematic review process, with changes being made after knowledge of the results of the individual trials. These findings relating specifically to Cochrane reviews indicate that even when stringent guidelines are in place and content experts are likely to be involved, adherence to good practice does not necessarily always follow. Additionally, Page and colleagues [18] found when analysing 682 systematic reviews (both Cochrane and non-Cochrane) that at least a third did not report use of a systematic review protocol. This means that substantial portions of systematic reviews are being conducted without making an accountable public record of the planned methods. Reporting guidelines are in place for systematic reviews in the form of the PRISMA statement [19, 20] to promote adherence to good practice, but again, uptake of compliance with guidance from systematic review authors is varied [7] and often poor. Wasiak and colleagues [21] report that, from a review of 60 systematic reviews in burn care management, 13 of the 27 PRISMA checklist items were addressed in less than 50% of cases. Whilst journals may stipulate to authors that the PRISMA checklist is followed when submitting systematic reviews for publication, peer reviewers and journal editors may be unlikely to find the time to monitor or regulate authors adherence to these guidelines, in addition to reviewing the scientific and reporting quality of the draft manuscript. The responsibility of maintaining good practice in systematic reviews should reside with the systematic review team. Awareness of relevant methodological guidelines and adherence to good practice may be more likely in a review team comprising of at least one experienced reviewer.

From protocol design to analysis, there are opportunities for team members to shape the project and these individual influences can affect the output [22]. Biases may be fairly obvious such as highlighting the most favourable results or perhaps more subtle and hard to detect such as biases in study selection (into the review or into the meta-analysis) towards positive studies. The motives of the review team can influence whether research findings represent true treatment effects and are able to be replicated [23] such as financial or other interests within the review team [12]. Ebrahim et al. [24] conducted a study of 185 meta-analyses in antidepressants to find that 29% of papers contained authors who were employees of the assessed drug manufacturer and that 79% had an industry link to the drug assessed. This study also found that meta-analyses including an author who was an employee of the manufacturer of the assessed drug were 22 times less likely to have negative statements about the drug than other meta-analyses. Additionally, Gómez-García et al. [25] assessed 220 reviews to investigate the role of the funding source in systematic reviews and meta-analyses in psoriasis. They report that reviews containing a high number of authors with conflicts of interest were of lower quality. Financial ”conflict of interest” (CoI) in systematic reviews has been shown to influence the results of published systematic review in the field of sugar-sweetened beverages and weight gain or obesity [26]. This evidence collectively suggests that a review team comprising individuals who are known to have a stake in the outcome of the review is a potential threat to the integrity of the report (sponsorship bias).

However, standard CoI statements often focus on narrow commercial interests and may be inadequate to reveal potentially hidden agendas [27]. Consumers of systematic reviews cannot rely solely on declarations of competing interests, which may relate to recent pecuniary funding (within the last 3 years), as opposed to more long-term affiliations to health interventions, to know whether those conducting the review have an interest in the results of the research (researcher allegiance) [28]. For example, for some, their very employment is reliant on a given intervention’s reputation such as homoeopaths or psychotherapists and they are unsurprisingly unlikely to publish a rigorous review with neutral or negative conclusions underpinning the basis of their profession. A recent study investigating the relationship between CoI and the conclusions of systematic reviews of psychological therapies found that “non-financial CoI” and particularly the inclusion of own primary studies into reviews were frequently seen in systematic reviews of psychological therapies [29]. Moreover, author allegiance to psychological therapy was never disclosed in 15 out of 95 reviews. Similarly, despite the apparent benefit that topic experts may bring to the review team, Gotzsche and Ioannidis (2012) [30] point out that the strong opinions of content area experts, such as clinical experts, can make it difficult to perform unbiased systematic reviews. For example, they assert that “people who have an interest in concealing uncomfortable evidence, clinicians, for example, find it particularly difficult to acknowledge the harms their interventions may cause*”*. Current methods for describing who is involved in the conduct of systematic reviews and their level of affiliation may occupy insufficient attention in final peer-reviewed journal publications.

Objective research, such as secondary evidence syntheses, should not necessarily be carried out dispassionately. The contribution of user perspectives and lay-expert engagement in research are now well-recognised [31]. Without patient and public involvement, due consideration to whether the review question or the included evidence are patient-centred may not be given [32]. In addition, global collaboration benefits the visibility and quality of scientific research activity [33], and failure to work collaboratively between institutions can have a *silo effect* on evidence synthesis projects supposedly deemed to be at the forefront of evidence-based medicine [34]. However, methodologists with particular skill sets who are not systematic reviewers such as statisticians and modellers employed in systematic reviews may be likely to recommend strategies which play to their strengths and that they have employed in the past and possibly without due consideration of other appropriate methods. Systematic reviews that fail to accommodate complex research questions may be at risk of being inflexible to useful innovations due to a reliance of “tried and tested” methodology [35]. For example, focusing on efficacy studies rather than pragmatic trials may jeopardise the external validity of review findings [36, 37]. Review teams preoccupied with critiquing heterogeneity of the existing evidence may be unlikely to elaborate on the external validity of the review, e.g., by searching and critiquing relevant grey literature, or service-user perspectives. Team experience or expertise could affect the external validity of a systematic review therefore. For example, research indicates that few strategies in the Cochrane Collaboration explicitly address the research priorities of disadvantaged populations and innovative approaches are needed to ensure that the research priorities of diverse stakeholders are considered [38, 39]. Equitable representation of population demographics within the team, as well as the more obviously required skills and experience may potentially influence the importance and uptake of the review.

In summary, there are a variety of factors that may influence the efficiency and appropriateness of the review team in addition to ensuring that the team comprises individuals with experience in systematic reviews. A well-trained methodological research workforce, continuing professional development, and involvement of non-conflicted stakeholders may help to ensure reproducible, accurate findings [2, 40]. But there are some less clear components underlying the nature and conduct of a review that can be influenced by motive, bias or error that may not be sufficiently addressed by current quality assessment tools for systematic reviews such as AMSTAR [41], GRADE [42, 43] and ROBIS [33] or explicitly evident from CoI declarations. Therefore, closer attention to who is conducting these secondary research projects, and why, is required rather than relying on the methods and conclusions of systematic reviews to speak for themselves. Systematic reviews are a powerful instrument in evidence-based medicine and are arguably more cost-effective than primary research [44]. But if systematic reviews are to maintain their primacy, their methodology should evolve through continued scrutiny in order to understand some of the (un)seen biases that can influence reviews from the review team composition, as well as the evidence that goes into them.

## Abbreviations

CoI: Conflicts of interest
